# Impact of bone-implant gap size on the interfacial osseointegration: an in vivo study

**DOI:** 10.1186/s12891-023-06215-1

**Published:** 2023-02-11

**Authors:** Kangkang Huang, Tingkui Wu, Jigang Lou, Beiyu Wang, Chen Ding, Quan Gong, Xin Rong, Hao Liu

**Affiliations:** 1 Department of Orthopedics, West China Hospital, Sichuan University, 37# Guoxue Lane, Chengdu Sichuan, 610041 China; 2 Department of Orthopedics, the First Affiliated Hospital of Zhengzhou University, Zhengzhou Henan, 450052 China

**Keywords:** Osseointegration, Cervical disc arthroplasty, Bone-implant interface, Bone defect

## Abstract

**Background:**

The bone-implant gap resulted from morphological mismatch between cervical bony endplates and implant footprint may have adverse impact on bone-implant interfacial osseointegration of cervical disc arthroplasty (CDA). The purpose of the study was to evaluate the impact of bone-implant gap size on the interfacial osseointegration in a rabbit animal model.

**Methods:**

A series of round-plate implants with different teeth depth (0.5 mm, 1.0 mm, 1.5 mm and 2.0 mm) was specifically designed. A total of 48 New Zealand white rabbits were randomly categorized into four groups by the implants they received (0.5 mm: group A, 1.0 mm: group B, 1.5 mm: group C, 2.0 mm: group D). At 4^th^ and 12^th^ week after surgery, animals were sacrificed. Micro-CT, acid fuchsin and methylene blue staining and hematoxylin and eosin (HE) staining were conducted.

**Results:**

At 4^th^ week and 12^th^ week after surgery, both micro-CT and HE staining showed more new bone formation and larger bone coverage in group A and group B than that in group C and group D. At 12^th^ week, the bone biometric parameters were significantly superior in group C when compared with group D (*p* < 0.05). At 12^th^ week, hard tissue slicing demonstrated larger portion of direct contact of new bone to the HA coating in group A and group B.

**Conclusions:**

Bone-implant gap size larger than 1.0 mm negatively affected bone-implant osseointegration between compact bone and HA coated implant surface.

## Introduction

Cervical disc arthroplasty (CDA) has been proven to be safe, effective and cost effective in the treatment of cervical degenerative disc disease (CDDD) [1, 2]. Even so, rate of implant dislocation was reported to range from 2.5% to 10.9% [3–5]. Such implant instability could cause severe consequences, including neurological disfunction and even paraplegia [6]. As for the long-term stability, good osseointegration is the key. Osseointegration is the direct contact of host bone to the implant without fibrous connective tissue to separate the two [7]. However, Lebl et al. [8] examined thirty retrieved Prodisc-C artificial discs (DePuy Synthes, West Chester, PA, USA) to find that mean bone ongrowth area was only about 7.2%. They also found that two of the six loosened implants showed no sign of bone ongrowth [8].

Many cervical artificial discs are designed to have flat footprint. Previous studies demonstrated that cervical bony endplates, especially the inferior endplates are concave in shape [9, 10]. We hypothesized that bone-implant gap might contribute to the poor interfacial osseointegration. Some studies on bone-screw interface supported this hypothesis that peri-implant bony defect was disadvantageous for osseointegration [11, 12]. However, these screws were inserted into cancellous bones, whereas implants were adjacent to cortical bones in CDA. Besides, it is unclear to what size the bone-implant gap would hinder the interfacial osseointegration. Therefore, in this study, different sizes of bone-implant gap between inner compact bone of the rabbit skull and the hydroxyapatite (HA) coated flat implant were established to mimic the bone-implant gap in CDA, in order to evaluate the impact of bone-implant gap size on interfacial osseointegration.

## Materials and methods

This in vivo study was approved by the Experimental Animal Ethics Committee of our hospital. A total of 48 male New Zealand white rabbits, aged 3 to 4 months old, weighted 2.5 kg to 3.3 kg, were used in this study. Only male rabbits were chosen because the oestrogen in females may affect the bone formation. All the animals were provided and accommodated by the Experimental Animal Center of our Hospital.

### Implant design and fabrication

**Fig. 1:**
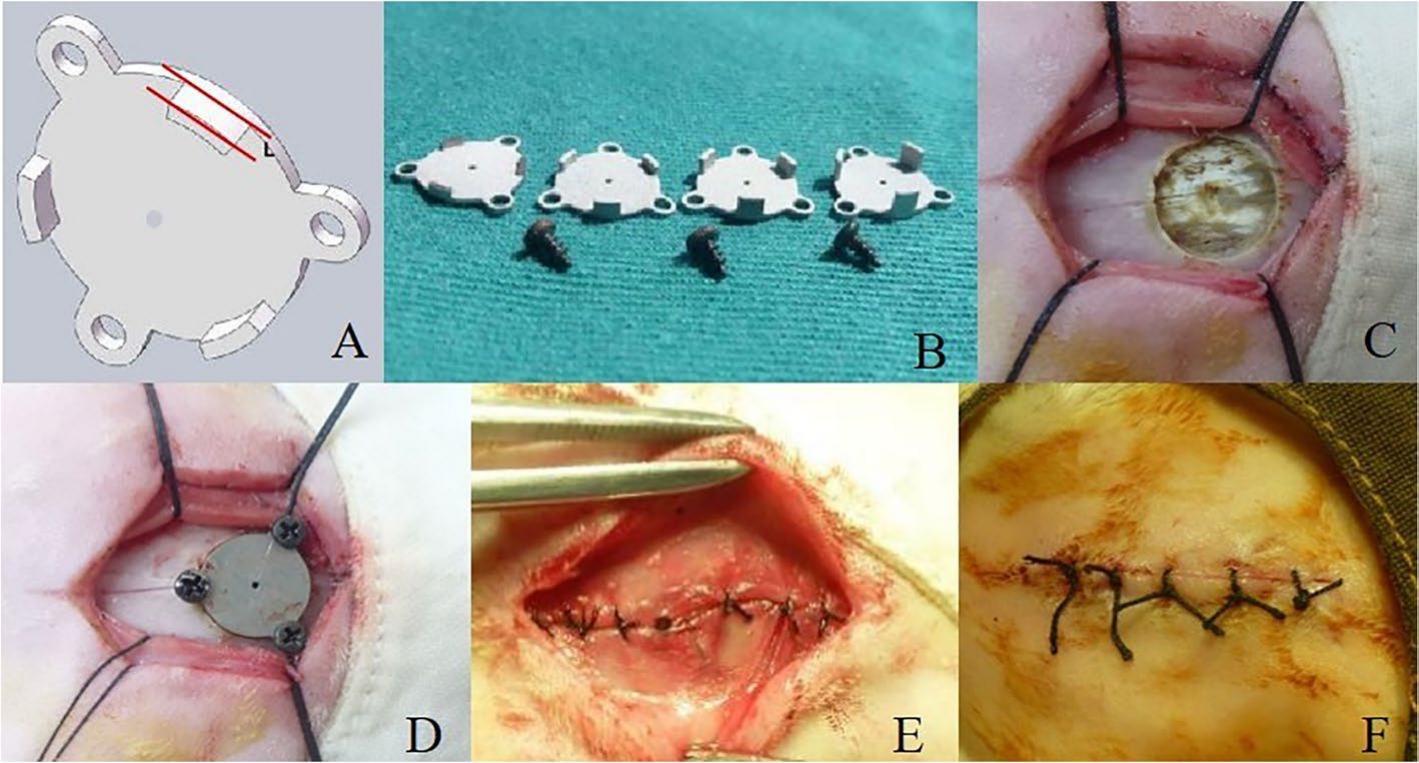
The implant used in our study and the surgical procedure. **A** three-dimensional model of the round plate implant. **B** Round plate implants with different teeth depth, from left to right, 0.5 mm, 1.0 mm, 1.5 mm and 2.0 mm. **C** A hole with the diameter of 8.0 mm was made (the depth was 0.5 mm in group A, 1.0 mm in group B, 1.5 mm in group C and 2.0 mm in group D). **D** Corresponding implant was fixed. **E** The aponeurosis was sutured. **F** The scalp was sutured

The implant was designed to be a round plate with 8.0 mm in diameter. Three teeth with different depths (0.5 mm, 1.0 mm, 1.5 mm and 2.0 mm, respectively) were designed to simulate the different bone-implant gap sizes. Three holes were added to the plate for screw fixation. The plate was made of Ti6Al4V. The inner surface of the plate was coated with HA, 59 μm in thickness and 58% in crystallinity. The implants were fabricated by CANSUN Ltd (Shandong, China) (Fig. 1 A and B).

### Surgical procedure

A total of 48 New Zealand rabbits were randomly assigned into four groups according to the different sizes of implant. The 3% pentobarbital sodium (1 ml/kg) was used for anesthesia. Hairs on the top of the skull was removed, followed by sterilization and draping. A 3 cm long midline incision was made at the top of the skull mid-way from the parietal bones. The aponeurosis was longitudinally dissected and retracted by suspending the four corners to properly expose the surgical field. Then a disc-shaped bur was used to create a round bony defects with continuous irrigation to decrease local temperature. The diameter of the bony defect was 8.0 mm. The depth was 0.5 mm in group A, 1.0 mm in group B, 1.5 mm in group C and 2.0 mm in group D. The outer compact bone and the intermediate cancellous bone were removed to expose the inner compact bone. Cautions were taken not to penetrate the inner compact bone. The surgical field was then irrigated before covering the bony defects with the plate implants with different sizes. Mini-screws were used to secure the implants to the skull. Penicillin (800 thousand unit) was administered intramuscularly for three consecutive days after surgery. The procedure had been showed in Fig. 1.

### Animal sacrifice

At the end of the 4^th^ week after surgery, 6 randomly selected rabbits in each group were euthanized with excessive pentobarbital sodium to obtain specimens for further evaluation (24 in total). The rest of the rabbits were euthanized with excessive pentobarbital sodium at the end of 12^th^ week after surgery (24 in total).

### Micro-CT analysis

For micro-CT image acquisition (Quantum GX, PerkinElmer, USA), following parameters were adopted: 80 kV of voltage, 500 μA of current, 3000 of exposure time, 14 μm of resolution, 200° of rotation angle, and 0.9° of rotation angle increment. The Scanco image system (Scanco Medical, Bassersdorf, Switzerland) was used to analyze bone tissues at the bone-implant interface. The region of interest (ROI) was determined as the cylindrical space within the round plate and teeth (π × R^2^ × H = π × 4 mm^2^ × 0.2 mm), where H was calculated from the HA surface of the plate toward the host bone. Following bone biometric parameters were obtained:

- Bone volume fraction (BVF)
- Trabecular number (Tb.N)
- Trabecular thickness (Tb.Th)
- Trabecular spacing (Tb.Sp)
- Tissue mineral density (TMD)
- Bone ongrowth area fraction (BOAF)

The BOAF was defined as the ratio of bone surface in direct contact with the implant to the total surface area of the implant surface.

### Histological preparation

For hard tissue slicing and acid fuchsin and methylene blue staining, specimens containing the implants and surrounding bone and soft tissues were fixed in 10% paraformaldehyde solution. After dehydration with ethanol solutions with escalating concentrations, specimens were embedded. Then specimens were subjected to slicing (SP1600, Leica, Germany), to get slices with thickness of 200 μm to 300 μm. Thereafter, each slice was subjected to grinding and polishing (E400CS, Leica, Germany), to finally get a slice with 50 μm. Then, slices were stained with acid fuchsin and methylene blue, and examined under light microscopy (DM4000M, Leica, Germany).

For hematoxylin and eosin (HE) staining, specimens were first fixed in 4% paraformaldehyde solution, followed by dehydration with ethanol solutions of escalating concentrations, and then embedded in resin. Thereafter, specimens were subjected to slicing (RM2235, Leica, Germany), to get slices with thickness of 6 μm to 10 μm. Slices were stained accordingly and examined under light microscopy (DM4000M, Leica, Germany).

#### Statistical analysis

Data were expressed as mean ± standard deviation and analyzed using SPSS software (version 19.0, IBM, Armonk, NY, US). Differences of means among different groups were compared by one-way ANOVA method after confirmation of data normality. Differences of means within each group between two time points were compared by *student-t* test. Difference was deemed statistically significant when *p* < 0.05.

## Results

At the end of 4^th^ and 12^th^ week, no difference in weight was observed among the four groups, before sacrifice and specimen harvesting. No implants displacement was observed.

### Micro-CT evaluation

**Fig. 2:**
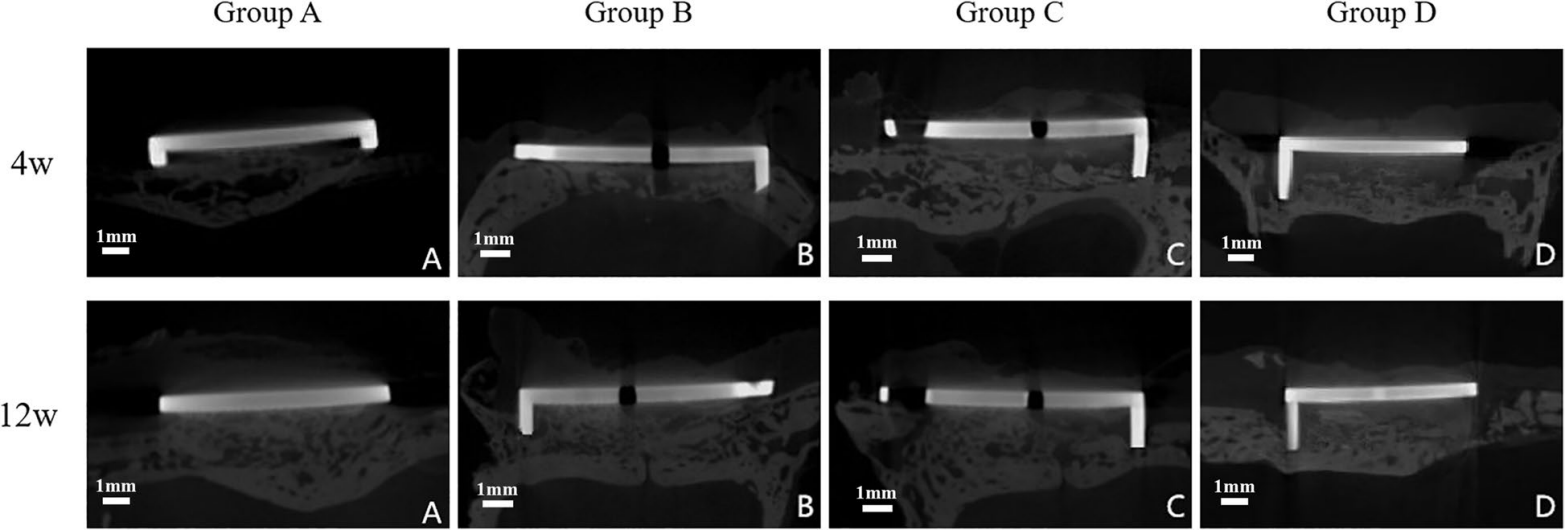
The outcomes of Micro-CT at 4^th^ week and 12^th^ week after surgery. At 4^th^ week, direct contact between bone and implant surface could be seen when bone-implant gap is 0.5 mm and 1.0 mm. Whereas evident interval could be seen when bone implant gap was 1.5 mm and 2.0 mm. At 12^th^ week, direct contact between bone and implant surface is achieved in when bone-implant gap is 0.5 mm, 1.0 mm and 1.5 mm. Whereas evident interval could be seen when bone-implant gap is 2.0 mm. **A** bone-implant gap = 0.5 mm. **B** bone-implant gap = 1.0 mm. **C** bone-implant gap = 1.5 mm. **D** bone-implant gap = 2.0 mm

In general, as depicted in Fig. 2, new bone formation and bone coverage were larger in group A and group B than that in group C and group D, both at 4^th^ week and 12^th^ week. In group C, the bone-free zone at the bone-implant interface was clear at 4^th^ week whereas bone coverage was achieved at 12^th^ week. In group D, however, bone coverage was poor at both 4^th^ week and 12^th^ week.

At 4^th^ week, no significant difference was observed between group A and group B, as well as between group C and group D, considering all bone biometric parameters assessed in this study. BVF, Tb.N, Tb.Th, TMD were all significantly larger in group A and group B than those in group C and group D (*p* < 0.05). Tb.Sp in group A and group B was significantly smaller than that in group C and group D (*p* < 0.05). At 12^th^ week, BVF, Tb.N, Tb.Th, TMD increased significantly whereas Tb.Sp decreased significantly within each group, compared with those at 4^th^ week. At 12^th^ week, no significant difference was observed between group A and group B considering all bone biometric parameters. BVF, Tb.N, Tb.Th, TMD were significantly bigger and Tb.Sp was significantly smaller in group C when compared with group D (*p* < 0.05). Results of bone biometric parameters are listed in Table 1.

**Table 1:** Bone biometric parameters obtained at 4^th^ week and 12^th^ week after surgery

		BVF (%)	Tb.N (mm^−1^)	Tb.Th (mm)	Tb.Sp (mm)	TMD (g/cm^2^)
Group A	4^th^ week	7.45 ± 1.46^*#^	3.45 ± 0.67^*#^	0.28 ± 0.05^*#^	1.88 ± 0.85^*#^	0.45 ± 0.04^*#^
	12^th^ week	16.47 ± 2.45^*#^	5.47 ± 0.75^*#^	0.42 ± 0.07^*#^	0.85 ± 0.45^*#^	0.66 ± 0.08^*#^
Group B	4^th^ week	6.73 ± 1.37^*#^	2.66 ± 0.56^*#^	0.21 ± 0.06^*#^	2.35 ± 0.96^*#^	0.38 ± 0.04^*#^
	12^th^ week	14.75 ± 2.13^*#^	4.82 ± 0.68^*#^	0.38 ± 0.06^*#^	0.98 ± 0.65^*#^	0.57 ± 0.07^*#^
Group C	4^th^ week	4.12 ± 0.87	1.36 ± 0.61	0.11 ± 0.08	3.56 ± 0.75	0.22 ± 0.03
	12^th^ week	8.83 ± 1.85^#^	3.46 ± 0.71^#^	0.22 ± 0.05^#^	2.10 ± 0.34^#^	0.40 ± 0.06^#^
Group D	4^th^ week	3.64 ± 0.79	0.85 ± 0.77	0.07 ± 0.10	4.45 ± 0.85	0.14 ± 0.06
	12^th^ week	6.12 ± 1.78	2.11 ± 0.85	0.13 ± 0.08	3.13 ± 0.65	0.28 ± 0.03

*BVF* Bone volume fraction (BVF), *Tb.N* Trabecular number, *Tb.Th* Trabecular thickness, *Tb.Sp* Trabecular spacing, *TMD* Tissue mineral density

^*^P < 0.05 when compared with Group C, # *P* < 0.05 when compared with Group D

**Fig. 3:**
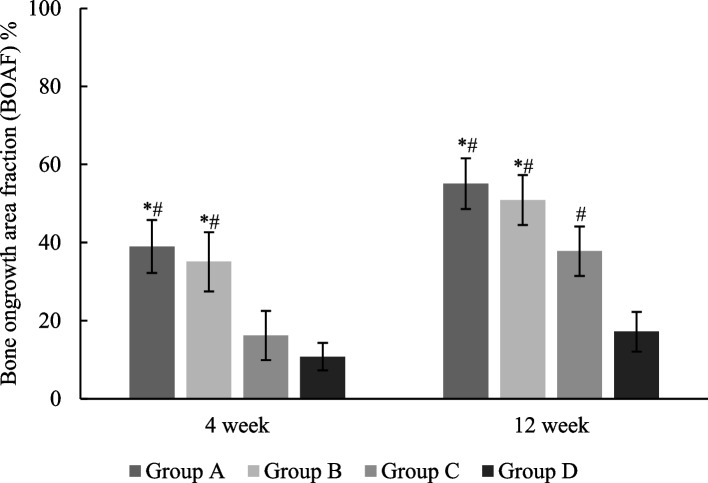
The bone ongrowth area fraction (BOAF) at 4^th^ week and 12^th^ week after surgery. At 4^th^ week, BOAF was significantly larger in group A and group B than that in group C and group D. At 12^th^ week, group D has the smallest BOAF, and group A and group B has significantly larger BOAF than group C. * *P* < 0.05 compared with group C. # *P* < 0.05 compared with group D

BOAF is showed in Fig. 3. At 4^th^ week, the BOAF in group A and group B (39.0% ± 6.8% and 35.1% ± 7.6%) were significantly larger than that in group C and group D (16.2% ± 6.3% and 10.8% ± 3.5%) (*p* < 0.05). The BOAF increased significantly in all groups from 4^th^ week to 12^th^ week. At 12^th^ week, the BOAF in group A and group B (55.1% ± 6.5% and 50.9% ± 6.4%) were significantly larger than that in group C and group D (37.8% ± 6.3% and 17.2% ± 5.1%) (*p* < 0.05). The BOAF in group C at 12^th^ week were significantly larger than that in group D (*p* < 0.05).

### Histological evaluation

**Fig. 4:**
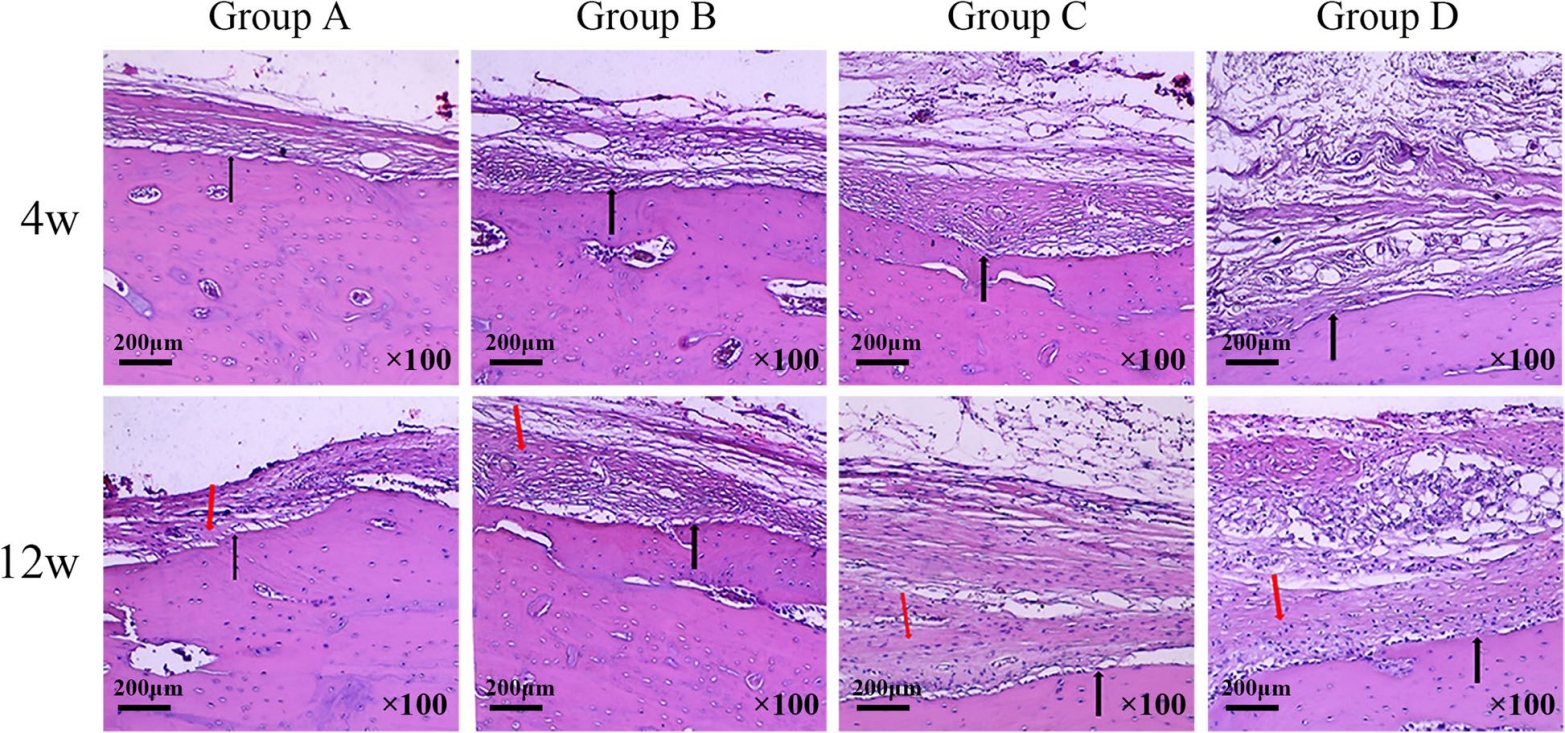
The hematoxylin and eosin (HE) staining of the specimens for the four groups at 4^th^ week and 12^th^ week after surgery. * The black arrow indicates the interface between mature bone tissue and newly formed bone tissue or fibrous tissue. The red arrow indicates the newly formed bone tissue

As showed in Fig. 4, the HE staining shows at 4^th^ week, newly formed bone tissue together with some fibrous tissue was seen in close contact to the host bone, in a relatively organized way, in group A and group B. Whereas in group C and group D, more disorganized fibrous tissue and less newly formed bone tissue were present. At 12^th^ week, more bone tissue could be seen in all four groups. In group A and group B, part of the newly formed bone tissue converted to mature bone tissue. In group C, more newly formed bone tissue with less fibrous tissue was present. In group D, fibrous tissue between the host bone and implant (not shown) was evident.

**Fig. 5:**
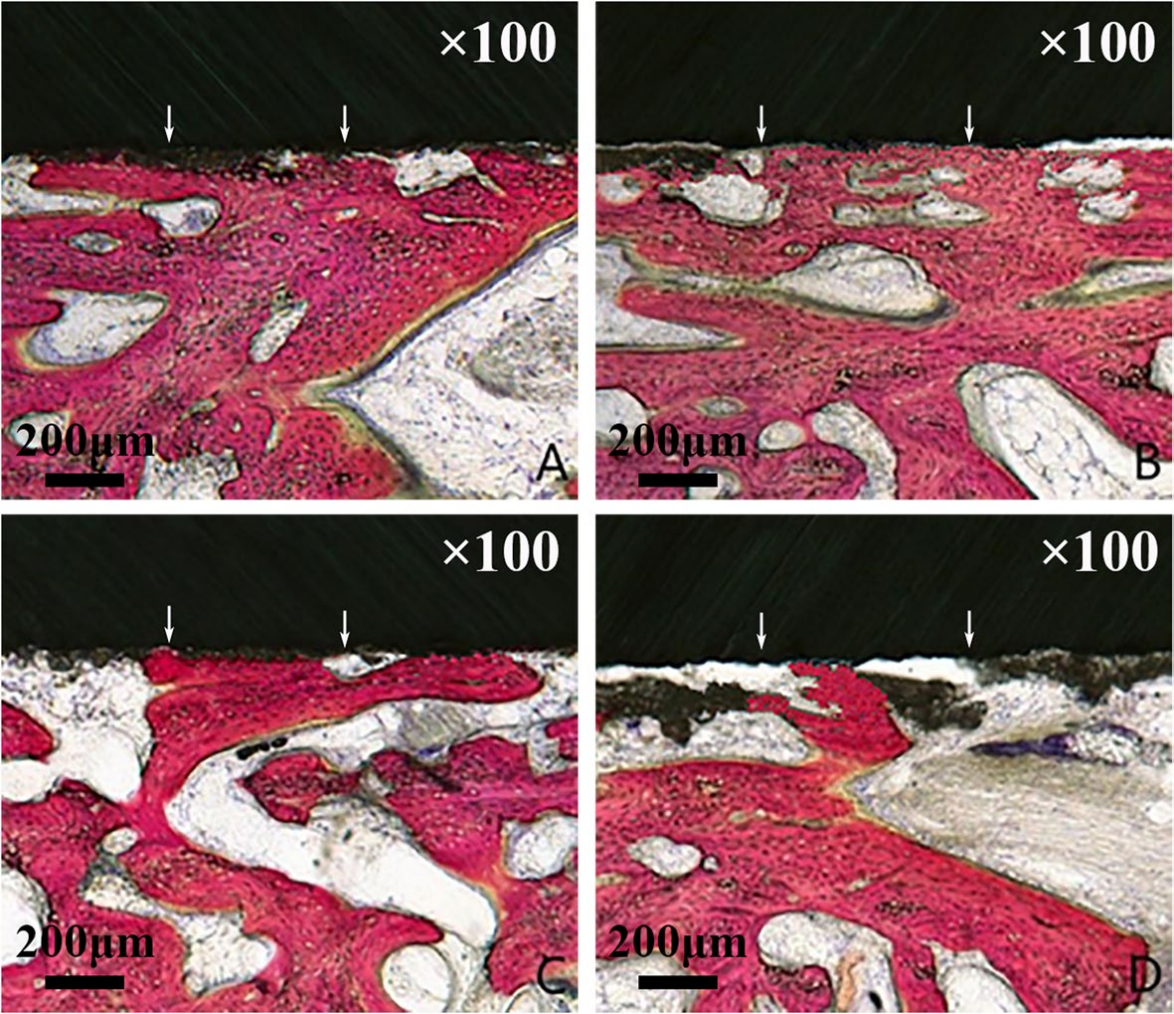
Acid fuchsin and methylene blue staining at 12^th^ week after surgery. Larger direct contact between new bone and the hydroxyapatite (HA) coated implant could be observed in group **A** and group **B** than that in group **C** and group **D**. The upper part of the picture (black) is the implant. The intermediate part (dark grey) is the HA coating. The lower part is the newly formed bone. * The white arrows indicate the interface between new bone and the HA coated implant

As shown on hard tissue slicing (Fig. 5), at 12^th^ week, more new bones (stained in red) could be observed in group A and group B, compared with that in group C and group D. In group A and group B, large portion of direct contact of new bone to the HA coating could be observed. In group C, less new bone was noted, as well as small portion of direct contact of new bone to HA coating. In group D, least new bone was formed and little portion of direct contact of new bone to HA coating was noted.

## Discussion

Many kinds of cervical disc prosthesis have been approved to be used in cervical spine surgery, each has unique design. The footprint of the prosthesis is either flat (Prestige LP, ProDisc-C, Discover) or dome-shaped (Mobi-C, ProDisc-vivo). Our previous study showed that cervical vertebral endplates are concave in shape with apex depth between 1.7 mm and 2.8 mm [9]. This mismatch between the shape of the prosthesis footprint and the cervical vertebral endplates created bone-implant gap. Some studies suggested that large bone-implant gap was one of the many risk factors for poor interfacial osseointegration [11, 12]. Although shaping the cervical bony endplate could enlarge contact area and reduce gap size between prosthesis footprint and bony endplate, thinned bony endplate might have lower strength to resist prosthesis subsidence [13]. Thus, it is important to maintain the balance between keeping the endplate integrity and reducing the bone-implant gap size. However, no consistence has been reached regarding to what size would bone-implant gap hinder the interfacial osseointegration. Sivolella et al. [12] evaluated the effect of bone-implant gap size on interfacial osseointegration in a canine mandible model. In control group the implant was in direct contact with bone, whereas in small and large defect group, bone-implant gap was 0.7 mm and 1.2 mm. The residual gap was 0.4 mm and 0.5 mm for small defect group and large defect group at 3^th^ month after implantation. Barros et al. [11] compared interface osseointegration of bone-implant gaps of 1.0 mm, 1.5 mm and 2.0 mm with that implant in direct contact with host bone (control group) in a canine mandible model. They concluded that wider gap showed worse osseointegration [11]. Interface osseointegration assessed by peri-implant bone density and bone-to-implant contact (BIC) were better in control group, 1.0 mm and 1.5 mm gap group, all significantly better than that in 2.0 mm gap group.

As mentioned above, together with many some other studies [14, 15], several bone-implant gap models were reported until now. Many researchers used screws, which were inserted into the pre-drilled, enlarged pit in canine mandible to evaluate the effect of gap size on interfacial osseointegration [11, 12]. In such models, bone healing process was exposed to intra-oral bacteria, and under shear-force caused by chewing at the bone-screw interface. This model could hardly represent the bone-implant interface in CDA. Some authors used a dumb-bell shaped implant, which was inserted into the femoral condyle [14, 15]. The dumb-bell shaped implant had uniformed gap size between individual animals. But the implant was in contact with cancellous bones. However, in CDA, the implant was in contact with cortical bony endplate. In this study, cylindrical (8 mm in diameter with different depth) calvarial bone-implant gap models were established to assess the impact of bone-implant gap size on interfacial osseointegration. The outer compact bone and the intermediate spongy bone were removed to create gap between the inner compact bone and the implant, to mimic the bone-implant interface in CDA. The disc-shaped bur and specifically designed plate implants made the gap comparable between individual animals within each group.

Results of this study showed that no significantly negative effect on the interfacial osseointegration was observed when bone-implant gap size was less than 1 mm. Direct bone contact can be seen in group A and group B at 4^th^ week after surgery, whereas in group C and group D little direct bone contact to the implant was seen. However, at 12^th^ week, bone contact between host bone and implant could be seen in group C. The possible theory is that, when gap size is less than 1 mm, new bone formation can take place at two frontiers [16]. On the side of host bone, osteoclasts and osteoblasts work together to form new bone toward the implant. On the side of the implant, osteoblastogenesis cells migrate to the surface by the scaffold formed by blood clot in the early phase. Under the effect of variant inflammatory cytokines and growth factors, these osteoblastogenesis cells proliferate and differentiate to mature bone cells to form new bone on the implant surface. However, bone-implant size exceeding 1 mm might hinder the cell migration to the implant surface. As shown in group C and group D, new bone formation could only be seen in the host bone side. Besides, when the gap size is too large, more than 1.5 mm for example in this study, might be prone to form fibrous connective tissue, rather than bone tissue, between the host bone and implant surface. Thus, as our results suggested, bone-implant gap size larger than 1 mm seemed to be bad for the interfacial osseointegration. The results were consistent with the clinical findings of our CDA patients which indicate that we should control the gap size in CDA or explore a biomaterial to fill the gap to improve the interfacial osseointegration.

Some limitations should be addressed. Firstly, the bone formation of the rabbit calvarial bone defect model was intramembranous ossification which may be different from the osseointegration of CDA endplate-implant interface. The results of our study should be verified in the endplate-implant gap model in the future. Secondly, the bone-implant gap in this study was almost cylindrical in shape, whereas the bone-implant gap in CDA was irregular. However, by using the specifically designed implants described above, bone-implant gap could be comparable within each group, to make the results more reliable. Thirdly, stress, micro-motion at bone-implant interface and the torsion mechanical testing was not able to be evaluated in this study. Theory that osteocytes were able to sense stress and orchestrate the process of osteoblastogensis and bone remodeling. Also, micro-motion within certain range was suggested to be positive in effect on bone growing. These factors and the interactions with bone-implant gap size need to be studied in the future researches.

## Conclusion

Bone-implant gap size larger than 1.0 mm could have negative impact on bone-implant osseointegration.

## Data Availability

Summarized data have been presented in this manuscript. The raw data for this study are located and protected at West China Hospital of Sichuan University. Sharing of the raw data is not suggested, because a secondary analysis is planned. The raw data could be obtained through contacting with the corresponding author (Hao Liu, Email: dr.liuhao6304@hotmail.com).

## Abbreviations

CDA: Cervical disc arthroplasty
CDDD: Cervical degenerative disc disease
HA: Hydroxyapatite
HE: Hematoxylin and eosin
BVF: Bone volume fraction (BVF)
Tb.N: Trabecular number
Tb.Th: Trabecular thickness
Tb.Sp: Trabecular spacing
TMD: Tissue mineral density
BOAF: Bone ongrowth area fraction
